# Early Optical Coherence Tomography Signs of Erdafitinib-Induced Retinopathy

**DOI:** 10.7759/cureus.66968

**Published:** 2024-08-15

**Authors:** Carson W Ercanbrack, Alexander S Kwok, Muhammad Z Chauhan, Shi M Tu, Sami Uwaydat

**Affiliations:** 1 Department of Ophthalmology, University of Arkansas for Medical Sciences, Little Rock, USA; 2 Department of Oncology, University of Arkansas for Medical Sciences, Little Rock, USA

**Keywords:** fgfr, fibroblast growth factor inhibitor, mekar, erdafitinib, chemotherapy-related toxicity

## Abstract

A 64-year-old male presented for a baseline ophthalmic exam before beginning erdafitinib, a fibroblast growth factor receptor (FGFR) inhibitor, for stage 4 papillary urothelial cancer. Baseline optical coherence tomography (OCT) and ophthalmic examination were unremarkable. After one month of treatment, his OCT demonstrated a significant thickening of the ellipsoid zone and prominence of the interdigitation zones along with a small amount of subretinal fluid. Two months after discontinuation of the medication, the OCT returned to baseline.

Erdafitinib is a Food and Drug Administration (FDA)-approved treatment for unresectable or metastatic urothelial cancer with FGFR2 or FGFR3 mutations. However, retinal toxicity can ensue with the initiation of the drug and cause subjective vision changes and OCT abnormalities. The drug may exert toxic effects on retinal pigment epithelium, which may be seen through interval OCTs and visualization of the interdigitation zone. Therefore, pronunciation of the ellipsoid and interdigitation zone on OCT may allow for surveillance of early manifestations of erdafitinib-induced retinal toxicity.

## Introduction

Bladder cancer is the tenth most common cancer on a global scale, with urothelial bladder cancer accounting for about 90% of the cases [1,2]. The five-year survival rate of metastatic bladder cancer in 2020 was only about 4.6% [1]. Various chemotherapy combinations exist but current first-line chemotherapy for metastatic bladder cancer is gemcitabine plus cisplatin or high-dose methotrexate, vinblastine, doxorubicin, and cisplatin plus granulocyte-colony-stimulating factor [3]. While up to 50% of urothelial cancers can respond to these first-line chemotherapies, mortality rates remain relatively high [3].

In 2019, the FDA approved erdafitinib as a chemotherapeutic agent that can be used in cases of susceptible urothelial cancers with FGFR2 or FGFR3 mutations that are unresectable or metastatic [4]. Fibroblast growth factor receptors (FGFRs) are tyrosine kinase receptors that, when mutated, can lead to carcinogenesis due to constitutive activation [5]. Erdafitinib is a pan-FGFR inhibitor that exerts its effects through competitive inhibition, therefore reducing the downstream effects of the pathologically hyperactive receptor [4]. However, retinal toxicity is a known side effect of the medication. As such, the FDA recommends a baseline ophthalmic exam prior to starting the medication, then every month for four months following the initiation of the chemotherapy, and every three months thereafter [6].

In this report, we document the baseline ophthalmic exam for a patient prior to beginning erdafitinib and the interval optical coherence tomography (OCT) following initiation of the drug. After one month of 8 mg of erdafitinib daily, the patient’s OCTs became altered. On discontinuation of the medication, his OCTs returned to baseline and his symptoms resolved. Thus, we propose that erdafitinib may induce retinopathy through disruption of the retinal pigment epithelium (RPE) and that early signs of toxicity may be able to be visualized with the elongation of the interdigitation zone (IZ) and ellipsoid zone (EZ) on interval OCTs.

## Case presentation

**Figure 1:**
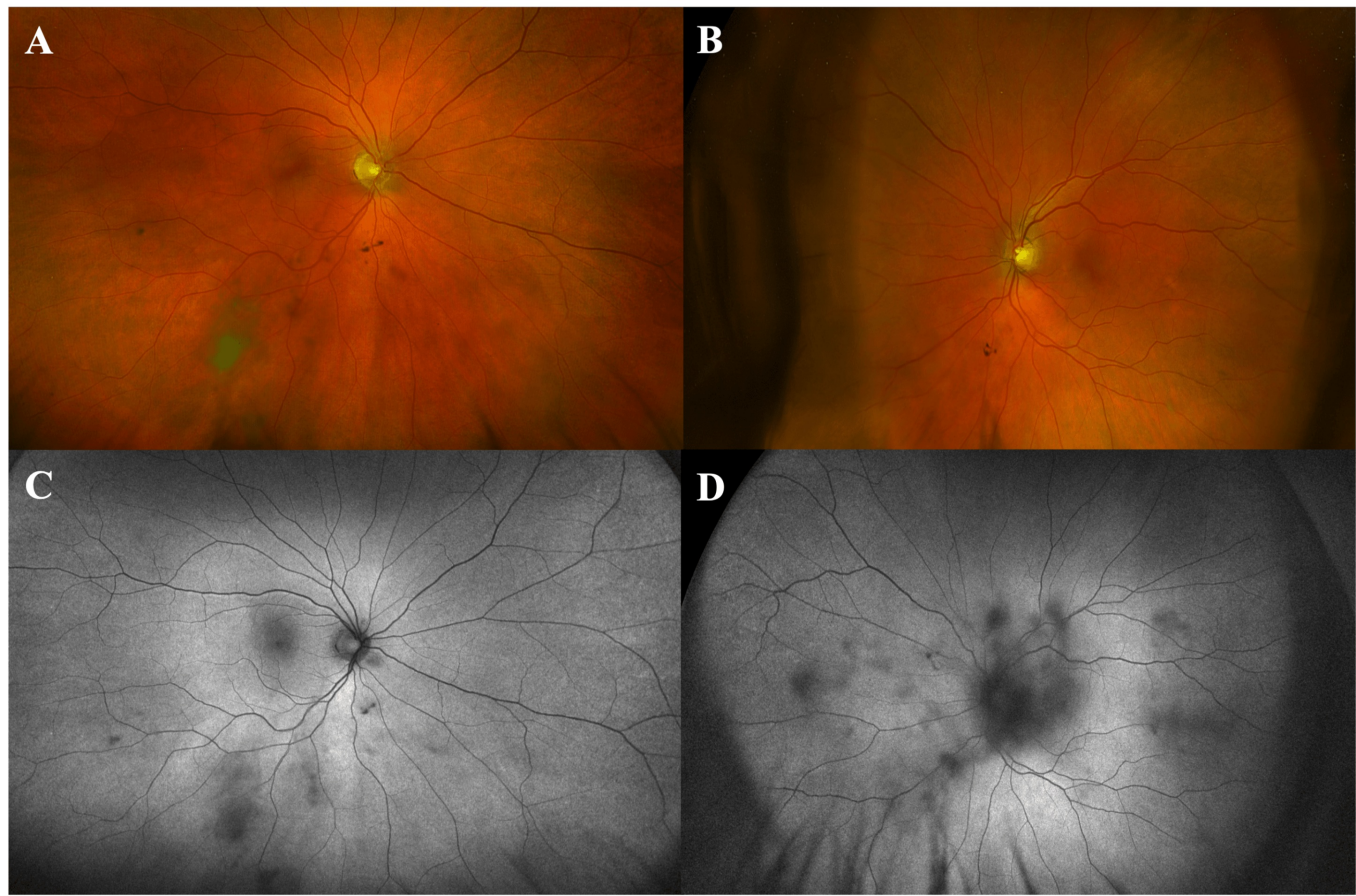
Optos pseudocolor, widefield fundus images and fundus autofluorescence prior to initiation of erdafitinib. Optos pseudocolor, widefield fundus images of the right (A) and left (B) eyes demonstrating normal fundus appearance with the exception of small vitreous opacities. Accompanying fundus autofluorescence images of the right (C) and left (D) eyes were also largely unremarkable outside of blocking artifacts from vitreous opacities.

**Figure 2:**
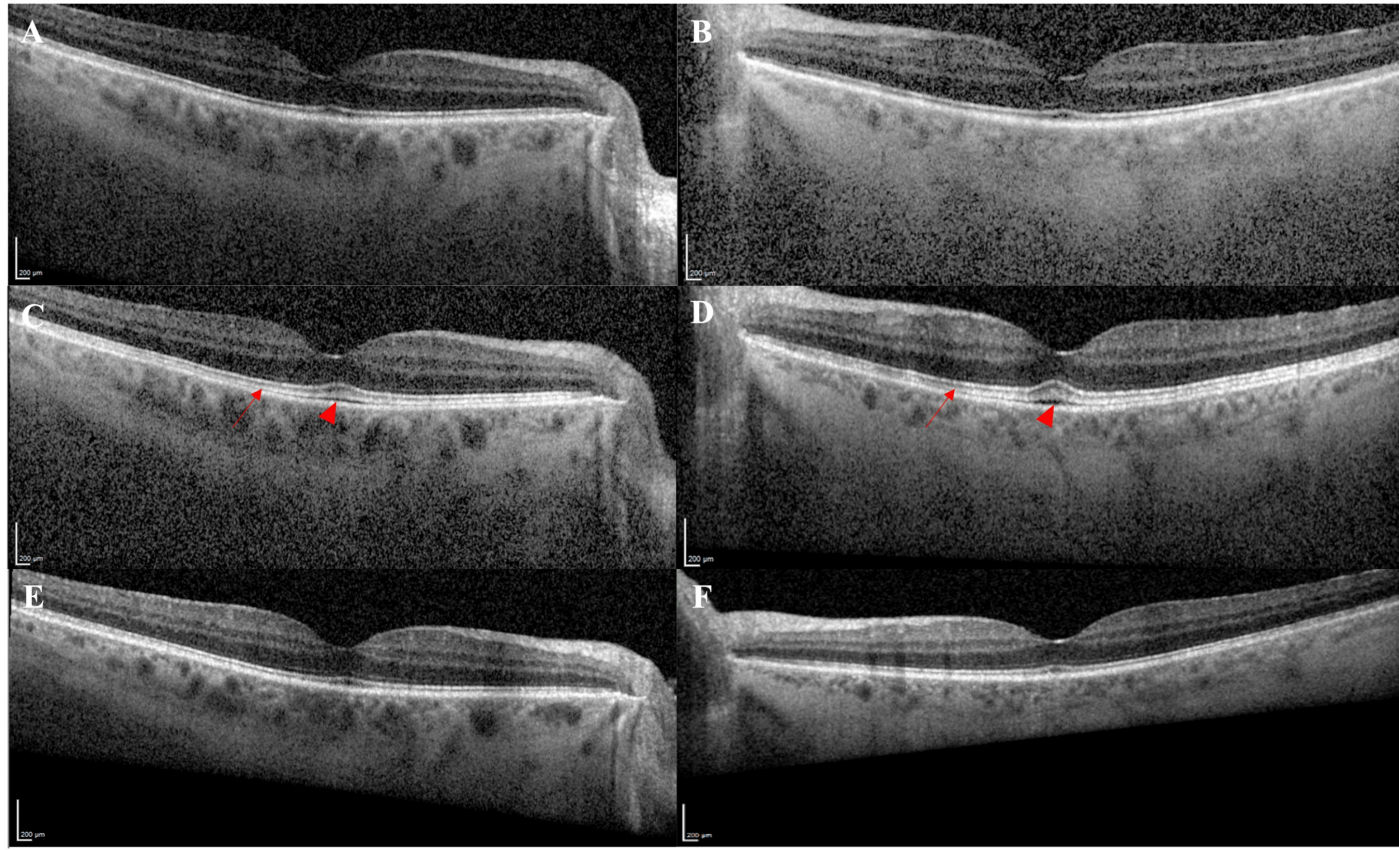
Optical coherence tomography images throughout the clinical course. Baseline optical coherence tomography (OCT) images of the right (A) and left (B) eyes, respectively, showing no gross abnormality before starting erdafitinib. One month after starting erdafitinib, the ellipsoid and interdigitation zones became significantly more prominent (arrow) with a small collection of subretinal fluid (arrowhead) in the right (C) and left (D) eyes. Separation of the photoreceptor outer segments from the retinal pigment epithelium and shaggy photoreceptor outer segments are an opacified line resembling a highlighter marking (C-D). Two months after discontinuation, the patient’s OCT images returned to baseline in the right (E) and left (F) eyes.

A 64-year-old man with stage 4 papillary urothelial cancer with positive FGFR3 mutation that was resistant to cisplatin/gemcitabine and pembrolizumab presented to the eye clinic for baseline eye examination before starting erdafitinib. His past ocular history included diabetes mellitus without diabetic retinopathy and pseudophakia in both eyes. The patient’s visual acuity (VA) was 20/20 in both eyes (OU) on the initial presentation. Anterior segment examination revealed intraocular lens in both eyes and posterior vitreous detachment. The remainder of the patient’s ophthalmologic exam, including Optos pseudocolor images and autofluorescence (Figure 1), was unremarkable. OCTs of both eyes showed normal retinal thickness without intra- or sub-retinal fluid (Figures 2A, 2B). The EZ to RPE apex measured 36 μm with central foveal thickness at 215 μm in the right eye (OD), and 34 μm with central foveal thickness at 217 μm in the left eye (OS).

The patient was started on oral 8 milligrams of erdafitinib daily within one week of the baseline eye exam. One month later, the patient presented with subjective blurriness of his vision along with visual disturbances. VA was 20/25 in the right eye and 20/20 in the left eye. The anterior and posterior segment exams were unchanged. OCT showed a significantly more prominent EZ, and the IZ was now well visualized. Both lines were now elongated with a highlighter-like appearance (Figures 2C, 2D). EZ to RPE apex now measured 71 μm with a central foveal thickness of 245 μm in OD and EZ to RPE of 85 μm with a central foveal thickness of 242 μm in OS. A small amount of subretinal fluid was also identified at this time. The patient reported onychomadesis, separation of the proximal nail plate from the nail bed, at this visit as well. The findings were communicated to the treating oncologist, who subsequently stopped the medication.

Two months following the discontinuation of erdafitinib, OCTs demonstrated normalization of the outer retinal layer (Figures 2E, 2F). EZ to RPE apex and central foveal thickness in both eyes returned to similar measurements before the administration of erdafitinib. The EZ to RPE apex was 20 μm with a central foveal thickness of 211 μm in OD and 35 μm with a central foveal thickness of 218 μm in OS. The patient reported that his vision was back to normal and VA at this time measured 20/20 in OU.

The treating oncologist resumed erdafitinib at a lowered dose of 6 milligrams daily three months after initial discontinuation. After two months of taking the lowered dose, the patient reported a recurrence of subjective blurriness of vision. OCTs at this time redemonstrated thickening of the IZ and EZ. Erdafitinib was subsequently stopped permanently due to the side effects and progression of the patient’s cancer at the lowered dose. The patient was referred for palliative radiation and passed away two months later.

## Discussion

**Figure 3:**
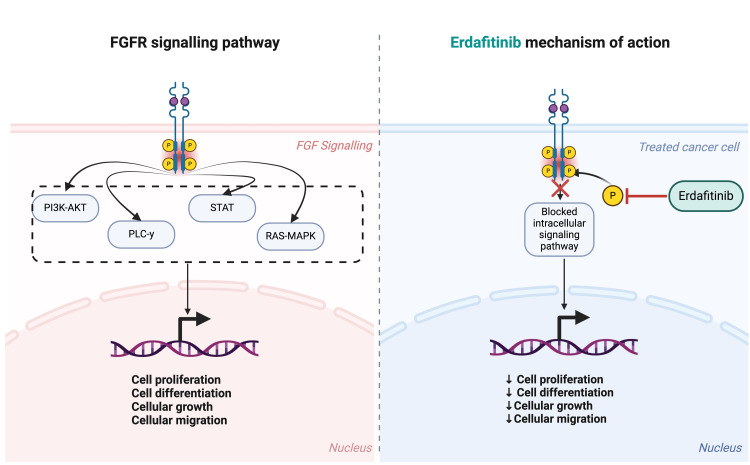
Diagram of fibroblast growth factor pathways and erdafitinib mechanism of action. Erdafitinib inhibits autophosphorylation of the fibroblast growth factor receptor (FGFR) cytoplasmic domain by competitively binding to the adenosine triphosphate (ATP)-binding site, controlling aberrant signaling through the RAS/RAF/MEK/ERK and PI3K/AKT pathways in cancers with FGFR genetic alterations. FGF: fibroblast growth factor; PI3K: phosphoinositide 3-kinase; PLC-y: phosphoinositide phospholipase C; STAT: signal transducer and activator of transcription; MAPK: mitogen-activated protein kinase; ERK: extracellular signal-regulated kinase. Figure created by Muhammad Z. Chauhan.

Erdafitinib (Balversa) is an FGFR inhibitor that was approved in 2019 for the treatment of unresectable or metastatic urothelial cancer that had FGFR2 or FGFR3 mutations. More specifically, erdafitinib prevents the phosphorylation of FGFR1-4, and, thus, acts as a tyrosine kinase inhibitor [7,8]. Ultimately, this suppresses the downstream responses of overactive FGFRs and reduces the anti-apoptotic effects of FGFR activation (Figure 3) [9]. Central serous retinopathy (CSR) and retinal pigment epithelium detachment (RPED) are known side effects of FGFR inhibitors [7,10].

The FDA recommends monthly ophthalmic exams during the first four months of treatment, and then every three months after that, or at the time of any visual symptoms [6]. The FDA also recommends withholding the medication if CSR or RPED occurs and reinitiating the medication at a lowered dose if CSR or RPED resolves within four weeks. If there is no resolution within four weeks, VA is equal to or worse than 20/200 in the affected eye, or if there is a recurrence of CSR or RPED after restarting the medication, permanent discontinuation of erdafitinib is recommended [6].

FGFR signaling occurs upstream from MEK in the RAS/RAF/MEK/extracellular-signaling kinase (ERK) pathway and plays a role in retinal maintenance, protection, and repair [11,12]. Therefore, the inhibition of FGFRs may produce a similar clinical picture as MEK inhibitor-associated retinopathy (MEKAR), consisting of bilateral fluid collections between the RPE and IZ [13,14]. Many of the adverse events associated with MEK inhibitors are caused by damage to the retina, with the most common being retinal vein occlusion and subretinal fluid accumulation [15]. The mechanism by which MEK inhibitor-associated retinal vein occlusion occurs is hypothesized to be caused by increased production of oxidative stress and inflammation and subsequent disruption of the blood-retinal barrier [15]. Subretinal fluid accumulation due to MEK inhibitors is proposed to be caused by damage to the RPE cells. MEK was found to regulate the tight junctions between the RPE via regulating the density of aquaporin 1 within the RPE [13]. Therefore, if RPE cells are unable to upregulate aquaporin channels adequately, fluid in the retina may be unable to be sufficiently transported outside the retina [15,16]. With a pan-FGFR inhibitor, additional pathways, such as the phosphoinositide 3-kinase (PI3K), B/AKT, Ras/mitogen-activated protein kinase (MAPK), and phosphoinositide phospholipase C (PLC-y) pathway are also prevented (Figure 3) [17]. The inhibition of these additional pathways may lead to the unique retinal pathology induced by a pan-FGFR inhibitor, such as erdafitinib, compared to that of MEKAR.

Fibroblast growth inhibitors are also toxic to the RPE [18]. RPE contributes to the blood-retinal barrier, phagocytoses the shed outer disks of the photoreceptors, and protects the retina from light-generated oxygen-reactive species [19]. Thus, early erdafitinib toxicity can result in the elongation of the photoreceptor outer segments due to the accumulation of the outer disks and can manifest on OCT by the increasing thickness of the IZ. Subretinal fluid can subsequently accumulate.

With the discontinuation of erdafitinib leading to symptomatic resolution and OCT return to baseline, the OCT abnormality seen after the patient began erdafitinib is likely due to the toxic effects of the chemotherapeutic agent on the RPE.

## Conclusions

Erdafitinib (Balversa) is a recently approved chemotherapeutic FGFR inhibitor used for metastatic or unresectable urothelial cancer. We believe that this case report is unique in two aspects. Firstly, the OCT findings normalized when erdafitinib was stopped, and promptly recurred when erdafitinib was resumed, demonstrating a positive rechallenge test. Secondly, the retinal toxicity in this case was dose-independent, as identical OCT anomalies recurred despite the lowered dose of erdafitinib. Current FDA guidelines recommend monthly ophthalmic exams in the first four months and then every three months after, or at the time of any visual symptoms to monitor for potential retinal toxicities associated with the drug. FGFR inhibitors can induce retinal toxicity possibly due to the disruption of the RPE, as evidenced by the elongation of IZ and EZ and new subretinal fluid accumulation on OCT after one month of treatment. The toxicity shares some similarities to MEKAR due to the FGFR inhibition occurring upstream from MEK. However, it is also unique from MEKAR in the fact that FGFR inhibition prevents the activation of other upstream pathways. After two months of discontinuing the drug, the patient’s OCT returned to baseline and the IZ and EZ were no longer elongated and the subretinal fluid had resolved. This furthers the idea that OCT changes were likely due to erdafitinib. Thus, we propose that the thickening of the IZ and EZ on OCT may allow for early detection of erdafitinib-induced RPE toxicity and allow for more tailoring of treatment.
